# Effectiveness of Telerehabilitation in the Treatment of Shoulder Injuries: A Systematic Review of Randomized Clinical Trials

**DOI:** 10.1016/j.arrct.2025.100553

**Published:** 2025-12-02

**Authors:** Danilo Santos Rocha, Edilaine Aparecida da Silva, Julia Tannus de Souza, Eduardo Lázaro Martins Naves

**Affiliations:** Department of Biomedical Engineering, Federal University of Uberlândia, Uberlândia, MG, Brazil

**Keywords:** Musculoskeletal disorders, Rehabilitation, Remote care, Shoulder injuries, Telerehabilitation

## Abstract

**Objective:**

To critically analyze evidence on the effectiveness of telerehabilitation in the treatment of shoulder injuries through a systematic review of randomized controlled trials.

**Data Sources:**

A comprehensive literature search was conducted in PubMed, Cochrane Library, Embase, and LILACS databases, covering studies published from 2019 to 2024.

**Study Selection:**

Studies were included if they were randomized controlled trials, published in English or Spanish, and focused on telerehabilitation interventions targeting shoulder injuries with outcomes related to pain and/or function.

**Data Extraction:**

Two reviewers independently selected and extracted data on study characteristics, participant demographics, intervention protocols, and reported outcomes. Disagreements were resolved by a third reviewer.

**Data Synthesis:**

Five studies involving 196 participants were included. Interventions varied in duration, frequency, and technological platforms. Most studies reported improvements in pain and function comparable to those achieved with in-person therapy, with high adherence and satisfaction rates.

**Conclusions:**

Telerehabilitation is a promising approach for managing shoulder injuries, providing effective, accessible, and personalized care. However, standardization of protocols and further research on complex pathologies are needed.

The impact of shoulder injuries on quality of life is crucial, as these conditions can cause chronic pain, functional limitations, and reduce the ability to perform activities of daily living.^1^ Additionally, recovery from shoulder injuries can be challenging, and in many cases, incomplete, which prolongs the negative impact on patients’ physical and emotional well-being.^2^^,^^3^ The need for prolonged treatments and the possibility of complications during the rehabilitation process also add layers of challenges, affecting not only shoulder function but also the mental health of the individuals affected.^4^

Shoulder injuries are among the most prevalent musculoskeletal disorders and encompass a wide range of conditions, such as rotator cuff tears, adhesive capsulitis, impingement syndrome, tendinopathies and shoulder instability.^5^^,^^6^ Each condition presents distinct pathophysiological characteristics, demanding specific rehabilitation protocols tailored to restore mobility, strength and function. For example, adhesive capsulitis involves progressive joint stiffness, whereas rotator cuff injuries lead to weakness and limited abduction, making individualized treatment crucial for optimal recovery.^1^^,^^7^^,^^8^

In this context, telerehabilitation has emerged as a transformative model in delivering physical therapy services, including therapy for shoulder injuries. Telerehabilitation refers to the use of information and communication technologies to remotely deliver rehabilitation interventions and monitor patients’ progress in real-time (fig 1). This allows patients to receive specialized care without the need for frequent travel to health care centers.^9^ This approach increases accessibility to rehabilitation services, especially in remote areas, and provides continuity of care, which is fundamental for effective recovery.^9^ This modality gained momentum during the coronavirus 2019 pandemic, highlighting the importance of remote care solutions in sustaining rehabilitation services and minimizing treatment interruptions.^10^

**Fig 1:**
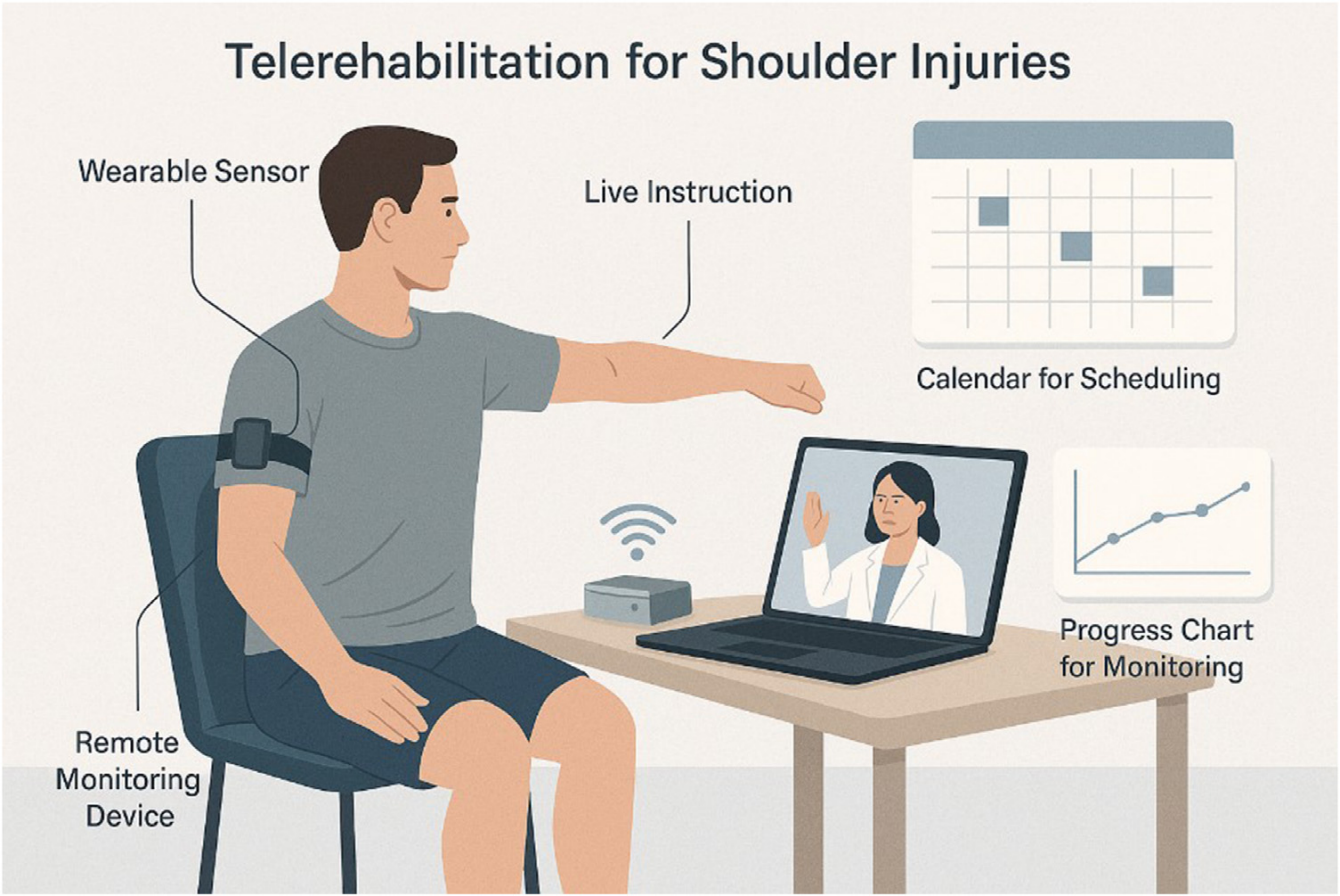
Telerehabilitation for shoulder injuries can use diverse technologies.

The benefits of telerehabilitation go beyond convenience. Its capacity for treatment personalization allows physiotherapists to design interventions based on patient-specific needs and functional goals, particularly relevant for shoulder conditions that often require nuanced and progressive rehabilitation.^11^^,^^12^ Telerehabilitation can also enhance treatment adherence, with patients feeling more engaged and motivated by incorporating technology into the rehabilitation process, improving the chances of long-term positive outcomes.^13^ Moreover, the use of real-time monitoring devices, mobile apps, and video-based consultations provides dynamic feedback and helps maintain therapist-patient interaction during the recovery process.^14^

However, telerehabilitation faces challenges such as comparing its effectiveness with traditional methods, technological adaptation by patients, and data security.^10^ Despite technological advances, issues remain, such as standardizing treatment protocols, adequately training health care professionals, and promoting patient acceptance of the method.^15^ Additionally, the heterogeneity of platforms and devices used for telerehabilitation may influence patient experience and treatment outcomes, indicating the need for further research to define best practices.^16^

This systematic review aims to critically analyze the available evidence on the effectiveness of telerehabilitation in treating shoulder injuries, highlighting key findings, research gaps, and prospects, providing a foundation to assist in the implementation and promotion of this innovative treatment modality.

## Methods

This study is a systematic review of randomized trials on shoulder telerehabilitation, registered in the International Prospective Register of Systematic Reviews (PROSPERO) under the registration number CRD42024564959 and is marked as completed. The systematic review was conducted in accordance with the Preferred Reporting Items for Systematic Reviews and Meta-Analyses (PRISMA) guidelines.^17^

### Search strategy

A comprehensive search was conducted in PubMed, Cochrane Library, Embase, and LILACS from January to May 2025**.** The search combined MeSH terms and free-text keywords related to “shoulder injuries” (including “rotator cuff,” “adhesive capsulitis,” “frozen shoulder,” “impingement syndrome”), “telerehabilitation” (including “tele-rehabilitation,” “telehealth,” “telemedicine”), and “efficacy” (including “effectiveness,” “outcome”), using Boolean operators (AND, OR) to refine the queries.

To enhance the comprehensiveness of the search and reduce the risk of missing relevant studies, synonyms were included in both English and Spanish, as these are widely used languages in the scientific literature, particularly in the field of rehabilitation and musculoskeletal disorders.

The PICOS framework was used to structure the search and define eligibility parameters, as summarized in table 1. As an observation, the term “in-person” was used to refer to therapeutic interventions delivered face-to-face, where the patient physically attends sessions at a clinic, hospital, or rehabilitation center under the direct supervision of a health care professional.

**Table 1:** PICOS strategy for literature search.

Component	Description
Population	Patients with shoulder injuries
Intervention	Telerehabilitation
Comparison	In-person rehabilitation
Outcomes	Functional improvement of the shoulder and pain reduction
Study design	Randomized controlled trials

### Eligibility criteria

The eligibility criteria used to select the articles included: (1) published article within the last 5 years (2019-2024) to focus on the most recent evidence and current practices in telerehabilitation; (2) English or Spanish language; (3) reported outcomes related to functional improvement and/or pain; (4) focused on telerehabilitation interventions targeting shoulder injuries; (5) be randomized controlled trials.

The exclusion criteria included: (1) interventions using in-person rehabilitation exclusively; (2) studies not focusing specifically on shoulder injuries; (3) studies inaccessible in full-text format.

### Study selection

The free web software Rayyan^a^ was used to remove duplicates and select eligible studies from the database findings. Two reviewers (D.R. and E.S.) independently selected the studies. Initially, the titles and abstracts of the articles found in the initial search were screened. Regardless of the study design (randomized controlled trials, cohort studies, or systematic reviews), clear criteria were established to include studies that examined the use of telerehabilitation in the treatment of specific shoulder injuries. After the initial selection, relevant articles were retrieved in full and assessed according to inclusion standards. When the 2 reviewers disagreed, a third reviewer (E.N.) was involved for discussion and consensus.

### Assessment of reporting bias and certainty of results

To ensure transparency and the robustness of the synthesis, rigorous methods were employed to assess reporting bias and the certainty of the results. The assessment of reporting bias was conducted by checking for the presence of missing or unreported results in the included studies. The potential for bias because of unpublished studies or selective reporting of results was investigated using tools such as the Assessing the Methodological Quality of Systematic Reviews, which helps detect publication bias. The certainty of the evidence was assessed using the Grading of Recommendations, Assessment, Development, and Evaluations system, which evaluates the confidence in the effect estimates based on criteria such as the consistency of results, risk of bias, precision of data, and applicability of findings. This approach ensured confidence in the final results and provided a solid foundation for the study’s conclusions and recommendations.

### Data analysis

To facilitate comparative analysis, pertinent data were extracted from the selected studies and organized into tables or spreadsheets. The information collected included study attributes (such as author, year, country), participant data (such as number, age range, type of shoulder injury), telerehabilitation intervention details (such as protocols, duration, frequency), and key outcomes reported (such as pain, physical function, quality of life).

Finally, the results were presented in a straightforward and understandable manner, following PRISMA (Preferred Reporting Items for Systematic Reviews and Meta-Analyses) guidelines. The clinical effects of the results, major research gaps, and suggestions for future research were discussed. This methodological approach ensured a transparent synthesis of the available evidence regarding the effectiveness of telerehabilitation for shoulder rehabilitation.

## Results

### Search results

The review began with the initial identification of 706 articles through searches in relevant databases. After removing 349 duplicates, the titles and abstracts of the remaining 357 articles were evaluated, resulting in the selection of 56 studies for full analysis. After thoroughly reading these studies, 51 were excluded for not meeting the inclusion criteria established for this review.

**Fig 2:**
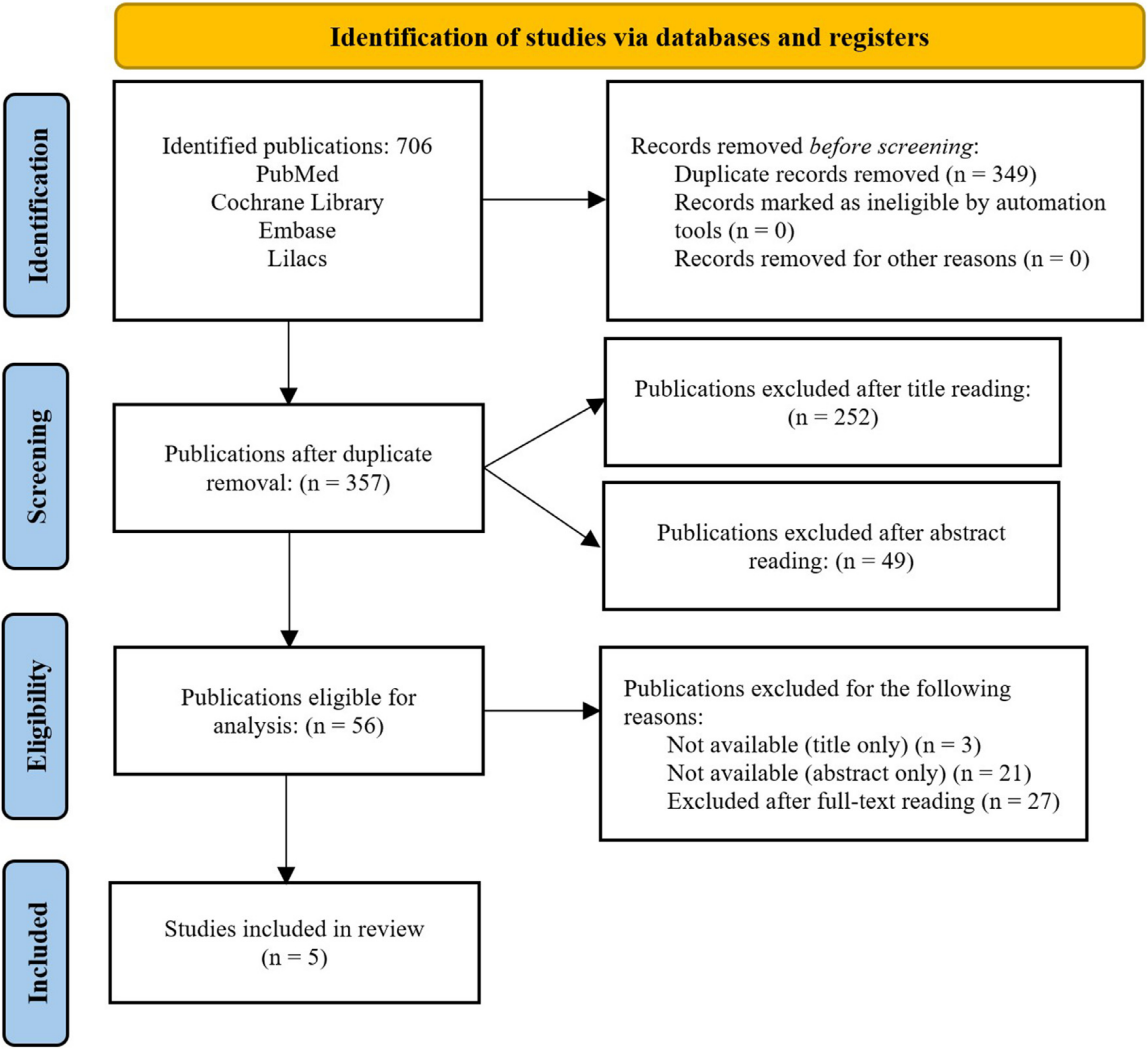
PRISMA 2020 flow diagram for the studies selection.

At the end of the selection process, 5 articles were considered suitable and included in this review because of their strict adherence to the inclusion criteria, thus providing a solid foundation for the analysis and synthesis of the results obtained in the research on shoulder telerehabilitation. The detailed flowchart of the study selection process can be viewed in figure 2.

### General data

The effectiveness of telerehabilitation in the treatment of musculoskeletal disorders has been extensively investigated in various recent studies, providing valuable findings on its clinical and economic benefits. This analysis of the results highlights the main findings and emerging trends of these investigations, offering a comprehensive view of the current state of shoulder telerehabilitation.

**Fig 3:**
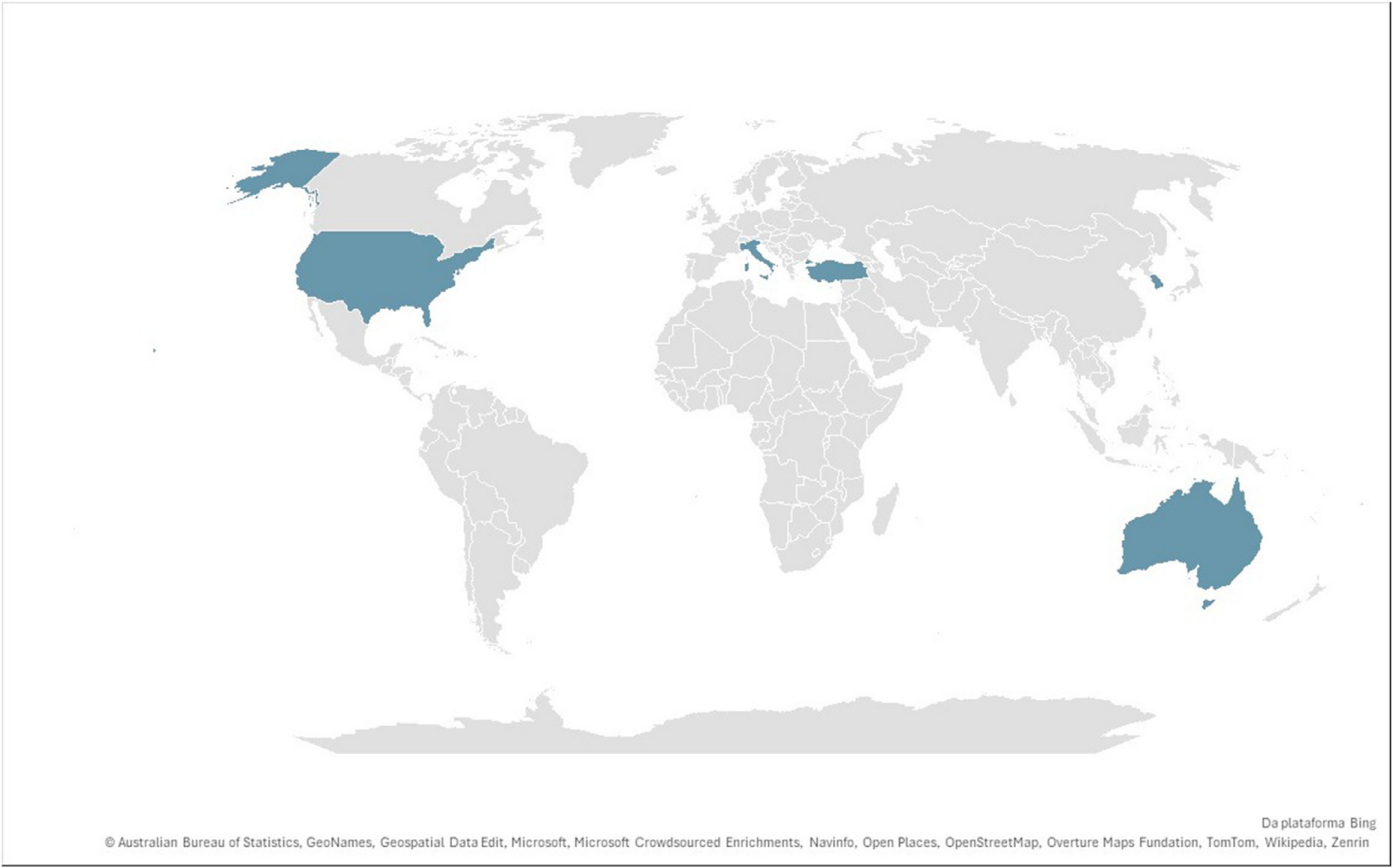
Country distribution for the selected studies.

The studies covered a total of 196 individuals distributed between intervention and control groups as indicated in table 2.^18^, ^19^, ^20^, ^21^, ^22^ Two studies included exclusively men^18^^,^^19^ whereas 2 studies involved samples of both sexes,^18^^,^^22^ and 1 study did not report the sex of the participants.^19^ Four studies assessed adults with pathologies related to the shoulder complex,^18^^,^^19^^,^^20^^,^^22^ and 1 study focused on children and adolescents with secondary complaints.^19^ The studies were from the following countries: United States, Italy, South Korea, Australia, and Turkey (fig 3).

**Table 2:** Characteristics of participants and exposure variables

	Sample			Exposure		
Author (Y)	Intervention/Control	Age and Sex	Comorbidities	Intervention	Control	Platform Used
Rizzato et al^18^ (2023)	11/11	Mean 61 y F=72%, M=28%	Impingement syndrome, capsulitis, tendon injuries, and degenerative pathologies	Digital therapy 40 min/d, 5×/wk for 2 wk	Equivalent nondigital rehabilitation program 40 min/d, 5×/wk for 2 wk	Playball
Pak et al^19^ (2023)	41/41	18-80 y (sex not reported)	Shoulder pain related to the tendon	Digital telerehabilitation 20 min/d, 3×/wk for 8 wk	In-person physical therapy 30 min/d, 2×/wk for 8 wk	Class II medical device
Jeong and Lee^20^ (2024)	18/19	10-20 y M=100%	Not reported	Diaphragmatic breathing, retraining, and shoulder stabilization exercises – telerehabilitation 3×/wk for 4 wk	Shoulder stabilization exercises – telerehabilitation 3×/wk for 4 wk	Zoom
Kenis-Coskun et al^21^ (2022)	10/9	6-15 y M=100%	Duchenne muscular dystrophy	Telerehabilitation – with monitoring 30-40 min/d 3×/wk for 8 wk	Home exercises – without monitoring 30-40 min/d 3×/wk for 8 wk	Laptop
Malliaras et al^22^ (2020)	12/24	51-56 y F=92% M=8%	Shoulder pain	Recommended care and telerehabilitation 3×/wk for 12 wk	n=12 only counseling; n=12 recommended care 3×/wk for 12 wk	Teleconference

Regarding the interventions, the study groups conducted training sessions with varying frequencies and durations reflecting a range of approaches. Four of the included studies^19^, ^20^, ^21^, ^22^ promoted training sessions 3 times a week, whereas 1 study adopted a frequency of 5 times a week.^18^ The duration of the sessions ranged from 20 to 40 minutes in 3 studies.^18^^,^^19^^,^^21^ However, 2 studies did not specify the duration of each session.^20^^,^^22^

**Fig 4:**
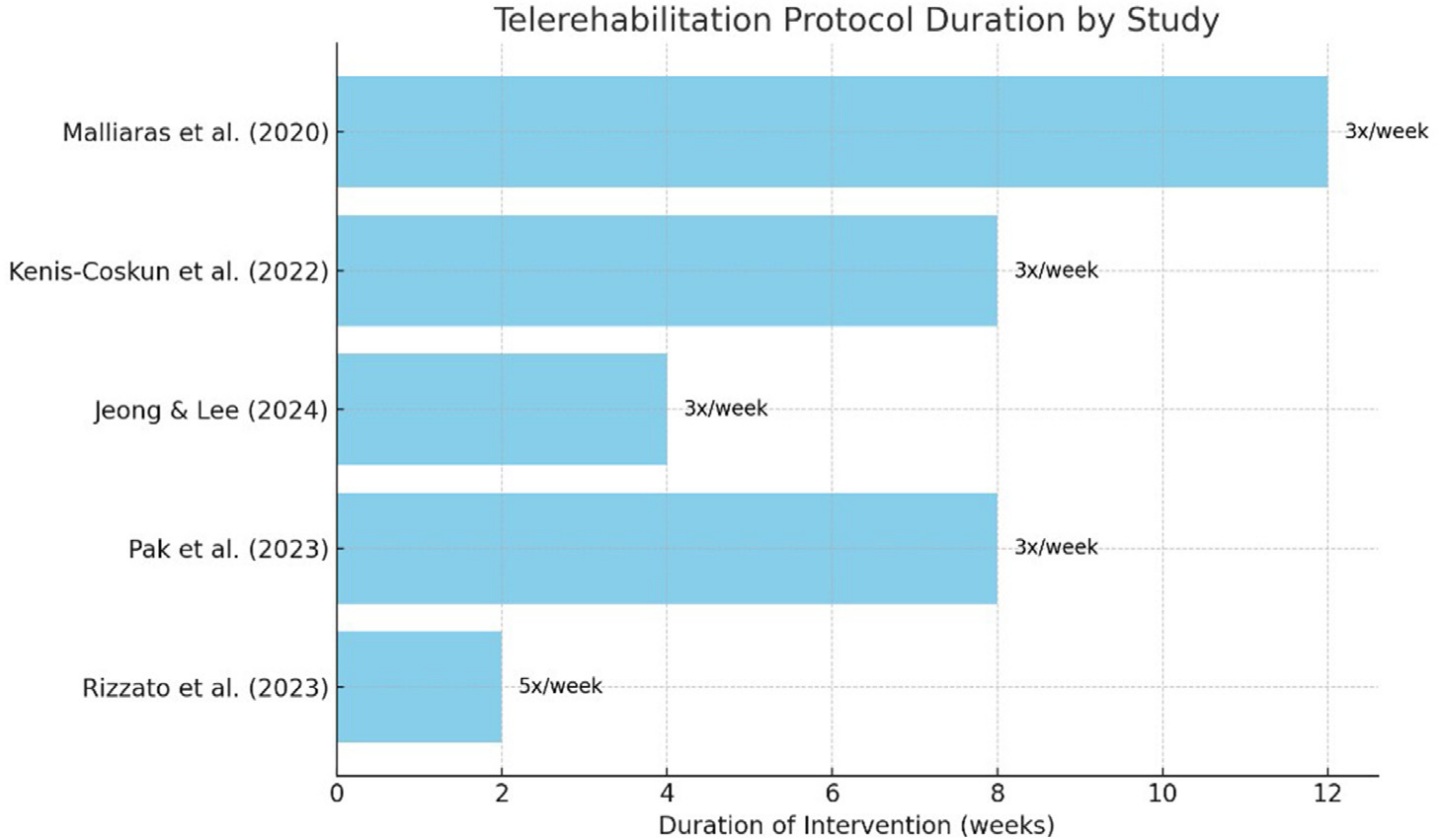
Telerehabilitation protocol frequencies per week and total duration by study.

The intervention protocols also differed in terms of total duration. Two studies followed an 8-week protocol,^19^^,^^21^ 1 study lasted 2 weeks,^18^ another lasted 4 weeks,^20^ and 1 extended over 12 weeks.^22^ Additionally, 2 studies maintained the same intervention time for both the intervention and control groups,^18^^,^^21^ while 1 study conducted interventions 3 times a week for the intervention group and 2 times a week for the control group^19^ (fig 4). Two studies did not explicitly compare the exposure time between groups.

The interventions varied in terms of approach. They included telerehabilitation,^18^, ^19^, ^20^ telerehabilitation with monitoring,^21^ and telerehabilitation with recommended care.^22^ These variations reflect the diversity and personalization of rehabilitation strategies used that aim to meet the specific needs of each participant group.

The studies used various platforms for telerehabilitation: Rizzato et al^18^ with Playball^b^, Pak et al^19^ with a class II medical device, Jeong and Lee^20^ with Zoom^c^, Kenis-Coskun et al^21^ with laptops, and Malliaras et al^22^ with teleconferencing systems, all essential for remote support and monitoring of the interventions.

The main conclusions of the studies analyzed in this systematic review are detailed in table 3.^18^, ^19^, ^20^, ^21^, ^22^ These conclusions are organized according to the hypotheses and objectives of each study, highlighting the key findings that emerged from the results of the interventions conducted.

**Table 3:** Main hypotheses, objectives, and conclusions of the studies.

Author (Y)	Hypothesis/Objective	Conclusion
Rizzato et al^18^ (2023)	Effectiveness of digital therapy using the Playball device during a shoulder rehabilitation protocol.	Greater engagement of the participant. Increased willingness to exercise individually at home after the supervised rehabilitation period.
Pak et al^19^ (2023)	Compare clinical and self-reported health outcomes between digital physical therapy and conventional in-person physical therapy in patients with chronic shoulder pain.	Digital programs can be viable models for providing care for chronic shoulder pain. High rates of acceptance, satisfaction, and adherence were observed in both groups, along with significant and comparable improvements in clinical outcomes.
Jeong and Lee^20^ (2024)	Investigate the effects of telerehabilitation combining diaphragmatic breathing retraining and shoulder stabilization exercises in young men with upper cross syndrome during the coronavirus 2019 pandemic over a 4-wk period.	Telerehabilitation with diaphragmatic breathing retraining and shoulder stabilization exercises improves neck pain, posture and function. Diaphragmatic breathing is effective for shoulder posture and balance in patients with upper cross syndrome.
Ozge Kenis-Coskun et al^21^ (2022)	Compare telerehabilitation with home video exercises regarding their effects on motor function, functional capacity, and muscle strength and overload.	A telerehabilitation approach for improving muscle strength is superior to a home video-based exercise program, but neither program improved functional outcomes in outpatients with Duchenne muscular dystrophy.
Malliaras et al^22^ (2020)	Evaluate the feasibility of a 12-wk internet-based intervention for rotator cuff repair surgical patients, comparing counseling only, recommended care, and recommended care with group telerehabilitation.	The prespecified success criteria were met or exceeded, but there was a sex imbalance (more women).

## Discussion

This review aimed to consolidate existing evidence on the effectiveness of telerehabilitation in the treatment of shoulder injuries, exploring the technologies used and the challenges and benefits of this therapeutic modality. Through the analysis of recent studies, we sought to provide a comprehensive and critical understanding of how telerehabilitation can be efficiently integrated into clinical practice, contributing to the functional recovery and quality of life of patients.

### Effectiveness of telerehabilitation for various shoulder pathologies

Evidence suggests that telerehabilitation is effective for various shoulder pathologies. For example, Huang et al^15^ conducted a meta-analysis comparing telerehabilitation and home exercises, concluding that both methods are comparable in terms of functional improvement and pain reduction. This approach not only offers convenience to the patient but also demonstrates significant clinical results, validating its application in physical health contexts. Additionally, the systematic review by Alahmri et al^5^ broadens this scope by evaluating the effectiveness of telerehabilitation for a wider range of musculoskeletal disorders, including shoulder issues, and concludes that this approach is effective in reducing pain and improving function. The results highlight telerehabilitation’s ability to address not only specific injuries but also chronic and acute conditions affecting shoulder functionality and patient quality of life. These findings are further supported by Vieira et al,^23^ who confirmed the efficacy of telerehabilitation across several musculoskeletal conditions.

However, some pathologies, such as chronic shoulder instability, still lack robust evidence regarding the effectiveness of telerehabilitation. Studies like Molina-Garcia et al^24^ indicate that while telerehabilitation is clinically effective, specific protocols for more complex conditions require further investigation to optimize outcomes. A detailed understanding of the mechanisms through which telerehabilitation exerts its beneficial effects is crucial for developing more targeted and effective interventions.

### Challenges and technological needs in implementing telerehabilitation

The successful implementation of shoulder telerehabilitation faces significant challenges, including the need for robust technological infrastructure and the ongoing adaptation of treatment protocols to meet individual patient needs. According to Rennie et al^25^ telerehabilitation is promising not only in reducing the costs associated with treating musculoskeletal disorders but also in improving access to care, especially in geographically dispersed or resource-limited areas. Cottrell et al^11^ highlighted that real-time telerehabilitation can produce outcomes comparable to traditional treatment, provided that digital access and clinical standards are maintained.

However, accessibility and digital competence also need to be addressed to ensure that all patients can equally benefit from this innovation. This includes the need for educational programs for patients and health care professionals on using digital technologies and the importance of digital literacy. Gava et al^26^ also emphasized the role of telerehabilitation in improving access and reducing disability in low-resource settings.

### Technological advances and remote monitoring

Technological advances, such as remote monitoring devices, have enhanced the effectiveness of home-based rehabilitation programs. Sassi et al^27^ highlight the importance of these devices in continuously supervising home exercises, providing real-time feedback and personalized adjustments as needed. This constant monitoring not only improves patient engagement but also optimizes therapeutic outcomes over time.^28^ Remote monitoring technology may include wearable sensors, mobile apps, and teleconferencing platforms that allow ongoing communication between the patient and therapist. Studies indicate that this continuous supervision can increase treatment adherence and improve long-term functional outcomes.^28^

### Augmented reality and interactivity in telerehabilitation

A notable advancement in telerehabilitation is the use of augmented reality, as explored by Yeo et al^10^ in their clinical trial protocol for patients with adhesive capsulitis. This study represents an innovative application of technology, using augmented reality to enhance patient interactivity and engagement during treatment. Although it is an initial protocol, preliminary results suggest potential for improving therapeutic outcomes in complex musculoskeletal conditions through more immersive and interactive approaches. Augmented reality enables the creation of virtual environments that can simulate therapeutic exercises and provide instant visual feedback, increasing patient motivation and movement accuracy.

### Guidance for clinical practice and future research

**Fig 5:**
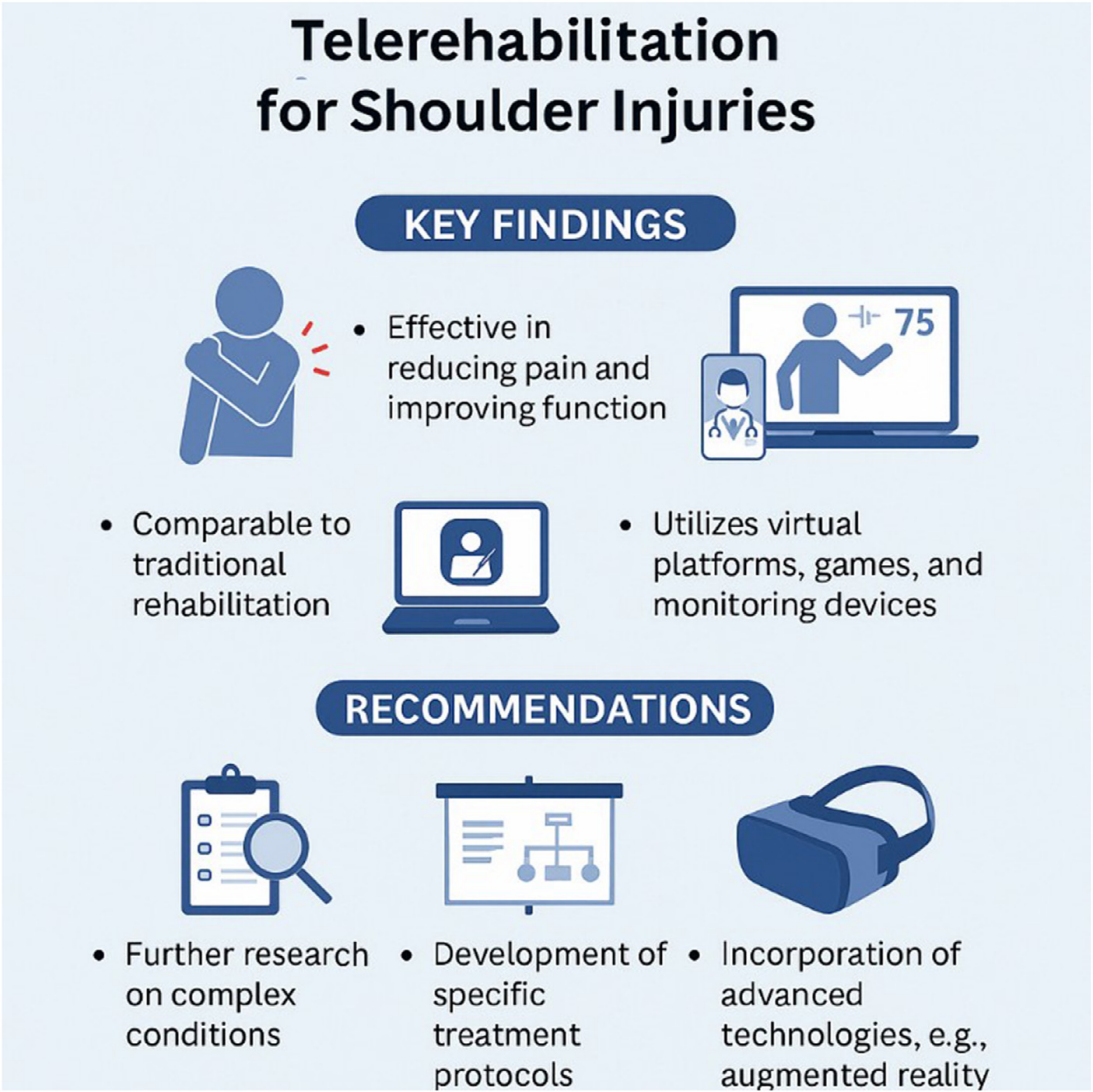
Key findings and recommendations from the selected studies.

To advance clinical practice in shoulder telerehabilitation, it is essential to invest in research exploring specific protocols for more complex conditions and using advanced technologies such as augmented reality. Developing evidence-based clinical guidelines and training health care professionals are crucial steps to maximize the benefits of this therapeutic modality. Pratama et al^29^ advocate for the inclusion of telerehabilitation in occupational rehabilitation programs to reduce musculoskeletal pain and improve functionality in workers.^29^ A summary of the key findings and recommendations from the included studies is presented in figure 5.

Additionally, it is important to develop and validate specific assessment tools to monitor treatment progression in telerehabilitation. This may include standardized questionnaires, functional performance assessments, and digital biomarkers that provide objective data on treatment effectiveness.

### Study limitations

It is important to recognize that the successful implementation of shoulder telerehabilitation faces significant challenges, including the need for robust technological infrastructure, data security, and the ongoing adaptation of treatment protocols to meet individual patient needs. Issues of accessibility and digital competence also need to be addressed to ensure that all patients can equally benefit from this innovation.

## Conclusions

The results of this review suggest that telerehabilitation can be a valuable tool for treating various shoulder pathologies, offering convenience and effectiveness comparable to traditional methods. Similar conclusions were drawn by Alahmri,^5^ who found telerehabilitation as effective as conventional physiotherapy for musculoskeletal disorders.

However, successful implementation requires overcoming challenges related to technological infrastructure, data security, and the adaptation of treatment protocols. Pak et al^19^ and Malliaras^22^ emphasize that digital models, when properly implemented, can achieve patient satisfaction and adherence rates comparable to or greater than traditional therapy.

To advance clinical practice in shoulder telerehabilitation, it is essential to invest in research that explores specific protocols for more complex conditions and utilizes advanced technologies such as augmented reality. Developing evidence-based clinical guidelines and training health care professionals are crucial steps to maximize the benefits of this therapeutic modality.

By considering these perspectives, it is possible to envision a future where telerehabilitation is more broadly and effectively integrated into clinical practice, improving access to treatment and quality of life for patients with shoulder injuries. Technological innovation, combined with a well-founded clinical approach, has the potential to transform physical rehabilitation and provide better outcomes for patients.

## Suppliers

a. Rayyan AI; Rayaan.
b. Playball; Playwork.
c. Zoom; Zoom Inc.
